# Treatment-resistant recurrent unipolar and bipolar depression: associative learning abnormalities

**DOI:** 10.1093/brain/awaf280

**Published:** 2025-08-04

**Authors:** Szabolcs Suveges, Yuxi Wang, Serenella Tolomeo, Tom Gilbertson, J Douglas Steele

**Affiliations:** Division of Neuroscience, Medical School, University of Dundee, Ninewells Hospital and Medical School, Dundee DD1 9SY, UK; School of Psychological and Cognitive Sciences, Peking University, Beijing, Beijing 100871, China; Institute of High Performance Computing, Agency for Science, Technology and Research, Singapore 138632, Singapore; Division of Neuroscience, Medical School, University of Dundee, Ninewells Hospital and Medical School, Dundee DD1 9SY, UK; Division of Neuroscience, Medical School, University of Dundee, Ninewells Hospital and Medical School, Dundee DD1 9SY, UK

**Keywords:** bipolar depression, recurrent depressive disorder, treatment-resistant illness, RLDDM, event-related fMRI

## Abstract

Severe and enduring psychiatric illness affects about 3% of the UK population and is associated with significant disability and a substantial reduction in average life expectancy. Two types are treatment-resistant recurrent unipolar depression and treatment-resistant bipolar depression, the latter being the depressed phase of bipolar disorder. Different phenotypes and different responses to antidepressant medications suggest different neural abnormalities. As bipolar depression can be clinically indistinguishable from unipolar depression yet require different treatments, it is important to develop objective ways to discriminate these two illnesses.

Here, we used reinforcement learning drift diffusion models of decision-making, and event-related fMRI acquired during a reward gain and loss avoidance task, to investigate patients with treatment-resistant recurrent unipolar depression and bipolar depression, in long-term General Adult Psychiatry follow-up.

We tested the null hypothesis that both unipolar and bipolar depressive illnesses show similarly blunted reward learning signals, and increased loss avoidance learning signals, with similar psychomotor slowing. Consistent with our null hypothesis, we found abnormally slowed decision-making for both depression types, with individual patient reinforcement learning drift diffusion model parameter estimates correlating with depression severity. For unipolar depression, we found blunted outcome and value signals for positive feedback, and increased signals for negative feedback. However, in contrast to our null hypothesis, bipolar depression was associated with preserved striatal reward prediction error signalling, and an absence of hippocampal and lateral orbitofrontal enhanced encoding of loss events, which was present for unipolar depression.

Overall, both treatment-resistant recurrent unipolar depression and treatment-resistant bipolar depression showed a similar pattern of neural abnormality compared with controls for the lateral orbitofrontal cortex reward value signal and the amygdala loss value signal. However, the illnesses also differed significantly, particularly with regard to hippocampal, striatal and lateral orbitofrontal function, potentially allowing objective discrimination. Using a support vector machine with the results of our neuroimaging analyses, it was also possible to differentiate the two depression types with an accuracy of 74.3%. Further studies of currently ill patients with severe and enduring illness are indicated.

## Teaser

Objectively distinguishing unipolar from bipolar depression is important but often difficult. Using brain scans and a decision-making task, Suveges *et al.* identify key differences in brain activity, suggesting that these two conditions can be reliably differentiated.

**See Pulcu (https://doi.org/10.1093/brain/awaf338) for a scientific commentary on this article.**

## Introduction

Severe and enduring psychiatric illnesses affect about 3% of the UK population and are associated with significant disability^1^ and an average reduction in life expectancy of 15 to 20 years.^2^ Two types of severe and enduring illnesses are recurrent unipolar depressive illness and bipolar depressive illness, the latter being the depressed phase of bipolar disorder. Treatment resistance is common with both, so patients are often in long-term follow-up by General Adult Psychiatry (GAP)^3^ services, which is where most UK psychiatrists work. The British Association for Psychopharmacology (BAP) consensus guideline for treatment of bipolar depression recognizes this illness is usually unresponsive to antidepressant medication^4^ in contrast to BAP’s consensus guideline for treatment of unipolar depression.^5^ As bipolar depression can be clinically indistinguishable from unipolar depression, but requires different treatments, there is considerable interest in identifying objective ways to discriminate bipolar depression from unipolar depression.^6-8^ Different clinical phenotypes, and different responses to antidepressant medication, may imply different neural abnormlaities.^4^

The US National Institute of Mental Health advocates conceptualization of studies using the Research Domain Criteria (RDoC) Matrix^9^ which links subjective symptoms to brain systems. RDoC is defined by different domains, such as the Positive Valence System (PVS) which processes reward information relevant to anhedonia, and the Negative Valence System (NVS) which processes information on aversive events such as losses.^9–11^ Anhedonia is a core feature of both types of depressive illness^12^ and blunted PVS striatal responses to rewards have been repeatedly reported for unipolar depression.^13–27^ Whilst there are fewer investigations of PVS abnormalities in bipolar depression, a review concluded that blunted striatal responses to rewards are not consistently reported,^27^ and a recent experimental study reported no blunting in bipolar depression.^28^ From a cognitive behavioural therapy perspective, increased attention to NVS-type aversive events, such as loss, are a core feature of both depression types,^29^ so it’s perhaps surprising that few neuroimaging studies of unipolar and bipolar depression have investigated loss events. Previously, we reported increased responses to losses in unipolar depression,^15^ and a recent study on bipolar disorder reported hyposensitivity to rewards compared with punishments in euthymic-recovered patients.^30^

The neuroimaging literature has additional limitations from the perspective of typical GAP patients. Neuroimaging and genetics studies of unipolar depression tend to be of mild non-treatment-resistant conditions, e.g. we recently published a large community-based neuroimaging study of 474 subjects^22^ of whom 72% were in the ‘no depression’ (healthy) range and 92% either in the ‘no depression’ or ‘mild depression’ range. Neuroimaging studies of bipolar depression, particularly moderate to severe treatment-resistant current illness, which investigate behavioural and neural responses to reward and loss events are rare.

Therefore, in the present study, our overarching aim was to test for the presence of behavioural and brain abnormalities in currently ill treatment-resistant patients with unipolar and bipolar depression in long-term follow-up by GAP services. Blunted responses to rewards and increased responses to losses are a core cognitive behavioural therapy conceptualization of unipolar and bipolar depression. PVS blunted reward prediction error signals and blunted encoding of reward value signals have been reported in many independent studies of unipolar depression, using reinforcement learning temporal difference models.^14,15,17,18,21,22^ We were interested in testing whether this pattern was present for currently ill, typical GAP patients in long-term National Health Service (NHS) follow-up, for both unipolar and bipolar depression. For these same patients, we were also interested in testing whether there was increased NVS loss prediction error signals and increased loss value encoding. These independent studies^14,15,17^  ^,18,21,22,31,32^ have highlighted the importance of specific regions for associative learning, so these regions were of *a priori* interest in the present study: the nucleus accumbens, ventral tegmental area, cortex, dorsal anterior cingulate, amygdala, hippocampus and hypothalamus. A recent review by Rolls et al. at Fudan University, has highlighted the importance of the lateral orbitofrontal cortex in unipolar depression,^33^ so we were particularly interested in testing for abnormalities in this region for both unipolar and bipolar depression.

Clinically, psychomotor disturbance, particularly slowing, has long been recognized in moderate to severe unipolar and bipolar depression^34^ with Widlocher^35^ and Parker^36^ proposing this as a core abnormality of moderate to severe illnesses. Recently, there has been increasing interest in modelling response times in unipolar depression using Drift Diffusion Models (DDM)^37^; however, these studies have not also investigated value learning using Temporal Difference Reinforcement Learning (TD-RL) models. Separate DDM-only and TD-RL-only models are limited: whilst the former explains only the decision-making process, the latter only captures how choices and outcomes are related to future decisions by estimation of values. Their combination, as Reinforcement Learning Drift Diffusion Models (RLDDM),^38-42^ offers a more comprehensive framework, allowing simultaneous testing of prediction error signal, value signal and DDM parameter hypotheses. To our knowledge, this approach has not yet been applied to the study of any depression type.

Using RLDDM modelling of behaviour and event-related functional MRI (fMRI), we tested the null hypothesis that both unipolar and bipolar depressive illnesses show similar blunted PVS reward learning signals compared with controls, and increased NVS loss avoidance learning signals associated with psychomotor slowing compared with controls.

## Materials and methods

### Participants

The study was approved by the Research Ethics Committee (10/S0501/20) and written informed consent obtained from all subjects. A total of 55 adults with treatment-resistant unipolar depression, treatment-resistant bipolar depression and controls were recruited, 17 patients with moderate severity bipolar depression [International Classification of Diseases (ICD) 10 F31.3], 18 patients with moderate severity recurrent unipolar depression (ICD 10 F33.1) and 21 controls. All patients had enduring illnesses requiring long-term follow-up over many years by GAP. Patients were recruited via the Advanced Intervention Service (AIS), an NHS national tertiary referral service hosted by NHS Tayside, which specializes in the assessment and treatment of treatment-resistant mood disorders. Exclusion criteria were any potentially confounding diagnosis including other primary psychiatric disorders, substance misuse or significant head injury.

For treatment-resistant unipolar depression, each patient’s clinical history was reviewed with regard to the number of failed adequate treatment trials in accordance with the Massachusetts General Hospital staging (MGH-S) method for unipolar depression.^43^ Typical MGH-S treatment-resistance ranges for NHS patients are: primary care 0.02–2.05, secondary care GAP clinic specializing in mood disorders 2.46–7.71, secondary care patients receiving Electroconvulsive Therapy 6.49–10.91, AIS Tertiary Care 9.36–16.84.^44^ As shown in Table 1, patients with recurrent unipolar depression were in the treatment-resistant range typical for Tertiary Care. In contrast to recurrent unipolar depression, no clinical consensus exists for the definition of treatment-resistant bipolar depression, although failure to improve following an adequate trial of two medications is a common theme.^45^ As noted earlier, the BAP consensus guideline recognizes that bipolar depression is usually unresponsive to antidepressant medication,^4^ in contrast to the BAP guideline for recurrent unipolar depression.^5^ All patients with bipolar depression had failed to respond to more than two adequate trials of medications, and all were in the depressed phase of their bipolar illness. Twenty-one healthy controls with no lifetime history of depression were recruited, mostly from partners, relatives and friends of the patients. None of the controls had any current or past psychiatric disorder and none were taking medication.

**Table 1:** Demographics and clinical characteristics

	Controls	Unipolar depression	Bipolar depression
Age, years	46.14 (13.63)	51.8 (10.94)	54.35 (10.99)
Female/total, *n*	15/21	15/20	14/17
National Adult Reading Test (NART)	6.3 (4.62)	6.4 (3.79)	7.79 (5.48)
Hamilton Depression Rating Scale (HAM-D)	0.48 (0.91)	16.1 (5.44)	9.88 (5.48)
Hamilton Anxiety Rating Scale (HAM-A)	0.43 (0.95)	15.65 (5.43)	11.29 (5.08)
Massachusetts General Hospital-Staging (MGH-S)	n/a	13.24 (10.78)	n/a

Depression severity was assessed using the 17-item Hamilton Depression Rating Scale (HAM-17)^46^ and anxiety assessed using the Hamilton Anxiety Rating Scale (HAM-A).^46^ Demographics and clinical characteristics are summarized in Table 1.

### Image acquisition

Functional whole-brain images were acquired for each participant with a 3 T Siemens Magnetom TimTrio Syngo MRI scanner using an echo-planar imaging sequence with parameters: repetition time = 2500 ms, echo time = 30 ms, flip angle = 90°, field of view = 224 mm, matrix = 64 × 64, 37 slices and voxel size 3.5 × 3.5 × 3.5 mm. To avoid transient effects the first four volumes were discarded.

### Behavioural task

**Figure 1:**
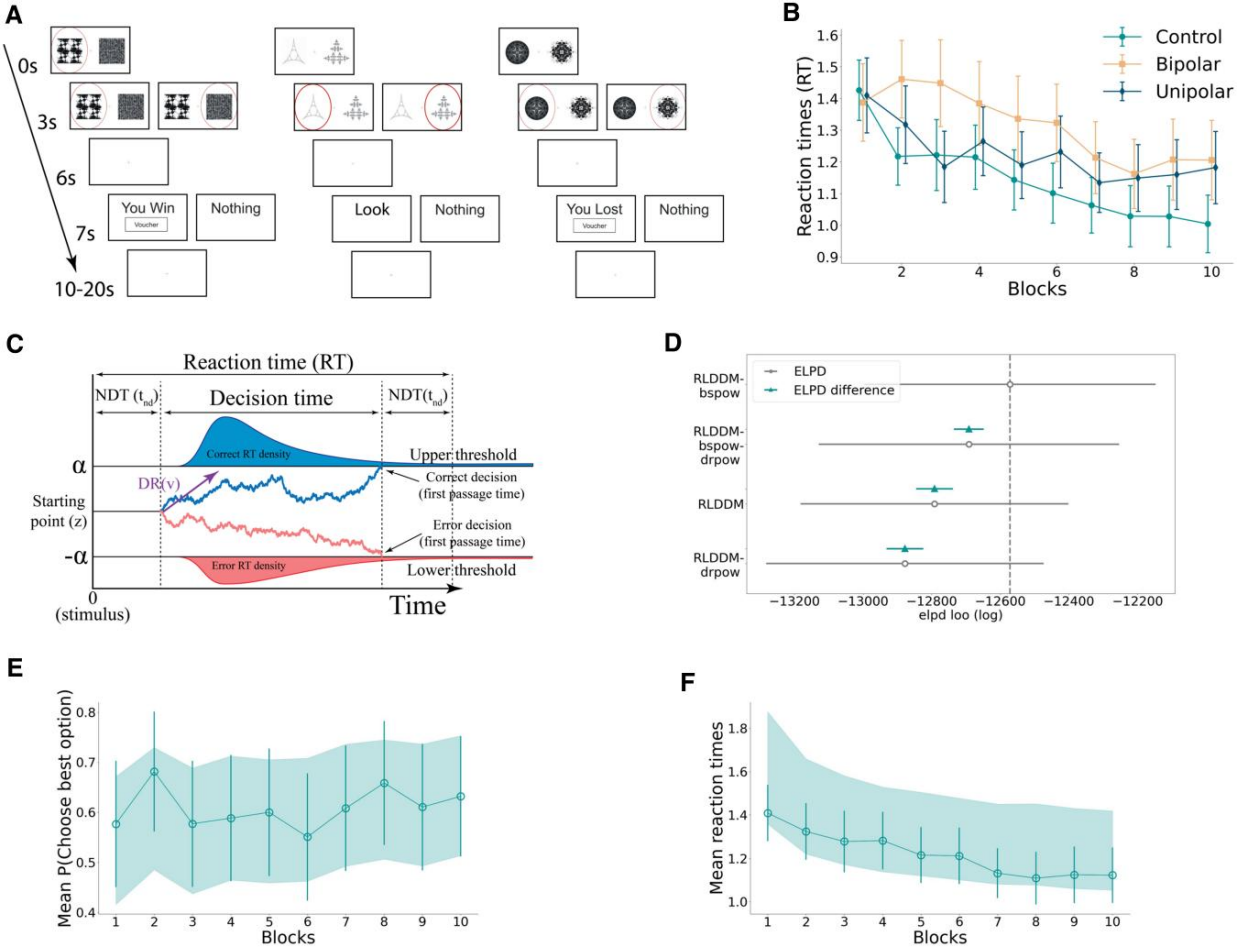
**Summary of task, computational model, model fit and checks.** (**A**) The modified Pessiglione behavioural task. (**B**) Time evolution of the reaction times for the three groups, where each block contains 18 trials for illustrative purposes. (**C**) Schematics of the Drift Diffusion Model with boundary separation a, non-decision time (NDT) t_nd_, z starting point and drift rate (DR) ν. (**D**) Model comparison of the four fitted models. Circles and triangles represent mean values, horizontal lines are the standard error of the mean (SEM), and the vertical dashed line is the mean elpd loo (log) value of the best model (RLDDM_bspow, see ‘Materials and methods’ section). Posterior predictive check for (**E**) the probability of choosing the best option and (**F**) reaction times where each block contains 18 trials for illustrating purposes. Each circle represents the mean of the observed variable, with vertical lines being the SEM and the shaded area being the 95% Highest Density Interval (HDI) of the generated data.

The behavioural task was modified from Pessiglione *et al*.^15,47^ with three types of outcomes, reward, neutral and loss. Each of type of trial had two outcome types: reward trials (‘you win’ or ‘nothing’), neutral trials (‘look’ or ‘nothing’) and loss trials (‘you lost’ or ‘nothing’). In contrast to the win and loss trials, participants’ choices on the neutral trials did not lead to any change in reward or loss value. Each trial type had 60 trials, totalling 180 for the whole task. One of the options/images in the reward (loss) fractal pairs had a fixed ‘high value’ 70% probability of winning (losing), and the other option had a fixed ‘low value’ 30% probability of winning (losing). Although the participants were not told these probabilities, they were told the goal of the task was to maximize winning and minimize losing ‘vouchers’ (‘points’) via trial and error. No payment was involved. Each trial began with the presentation of one of the fractal pairs, with one randomly assigned to the left of the screen and the other to the right of the screen. Participants had to choose between the two presented images in under 3 s, after which a fixation cross was displayed for 3–13.75 s, a variable duration optimized for signal detection using the optseq2 algorithm,^48^ and finally a feedback outcome was shown. A summary of the task is shown in Fig. 1A.

### Computational modelling of behaviour

The RLDDM captures both the subject’s choices and their reaction times^40,41^ which was considered particularly relevant for testing hypotheses about treatment-resistant unipolar depression and bipolar depression. This is because it incorporates modelling of reaction times and therefore psychomotor slowing whilst retaining the advantages of the temporal difference method^49^ for value learning.

RLDDM involves two main parts: one that describes the update of value expectations and a part accounting for decision-making. The former is achieved using the temporal difference method where we update the value Q(o, t) of the chosen option o at trial t by:


(1) Q(o,t)←Q(o,t)+α(R(t+1)−Q(o,t))

where *R*(*t* + 1) denotes the reinforcement at trial t + 1, 0<α<1 is the learning rate and *R*(*t* + 1) − *Q*(*o*,*sst*) is the prediction error signal.

The decision-making part is described by a DDM illustrated in Fig. 1B with the rate of evidence accumulation or drift rate *v*(t) at time *t* described by:


(2) $$\nu (t)=(Q_{upper}(t)-Q_{lower}(t))\times \nu$$

where *Q*_upper_(*t*) (*Q*_lower_(*t*)) is the expected value of the upper (lower) decision threshold, and *ν* is a free scaling parameter associated with the degree of exploration/exploitation. Hence, drift rate is scaled by the difference between the higher (*Q*_upper_(*t*) = *Q*(high value option, *t*)) and lower value option estimates (*Q*_lower_(*t*) = *Q*(low value option, *t*)) at trial *t*. Using the Wiener First Passage Time (WFPT) distribution, the likelihood of a reaction time RT(*o*, *t*) of a choice *o* at trial *t* is given by:


(3) $$RT(o,t)WFPT(a,t_{nd},z,\nu (t))$$

where the boundary separation *a* and non-decision time *t_nd_* are trial-independent free parameters, and *z* is the starting point that we fixed (z = 0.5) as we randomized the side of the screen on which pictures were presented to subjects.

We explored two extensions of this RLDDM model,^41^ one that allowed the drift rate and the other that allowed the boundary separation to vary between trials via a power function:


(4) $$\nu (t)=(Q_{upper}(t)-Q_{lower}(t))\times {\left(\frac{t}{10}\right)}^{p}$$

(5) $$a(t)=bb\times {\left(\frac{t}{10}\right)}^{bp}$$

Here, *p* and *bp* are free parameters controlling the direction of the time evolution of the drift rate and boundary separation parameters, respectively, and *bb* is the boundary separation baseline, a free parameter.

Using these two modifications, we created four different models: a base model without any addition (RLDDM), a model with a drift rate modification Equation (4) (RLDDM_drpow), a model with a boundary separation modification Equation (5) (RLDDM_bspow) and a model with both modifications (RLDDM_bspow_drpow).

For all models we assumed that the model variables did not vary between trial types (reward, loss and neutral), but the value estimates did, except for neutral trials where we fixed $Q_{upper}(t)-Q_{lower}(t)=0.4$, the difference between the contingencies, which was necessary to ensure our fitted models passed all convergence tests.

### Model fitting

We fitted these models to our behavioural data using a two-level hierarchical Bayesian modelling approach within Stan (CmdStanPy interface version 1.24)^50^ using an existing implementation,^38,39,51^ with four sampling chains, each with 1000 (discarded) warm-up and 3000 iteration samples. To access the convergence of the fits, we computed using the Arviz package,^52^ the estimate of rank normalized split Gelman–Rubin statistic (Split-$\hat{R}$),^53^ as well as the tail effective sample size (tail-ESS).^53^ All our model fits met recommended thresholds for both of these criteria, Split-$\hat{R}$ < 1.01 and tail-ESS >400. We also performed posterior predictive checks for both choices and reaction times. Using the samples of the model parameters, we generated synthetic choices and reaction times and found that these overlap with the experimental data, implying a good model fit that captures the behavioural decision-making data (see Fig. 1D and E for winning model).

### Model comparison

To compare the models, we used 5-fold cross-validation and calculated the Expected Log-Predictive Density (ELPD) for each model using the difference of the ELPD values between models and the standard error of these differences,^54^ finding that RLDDM_bspow was the best model, as seen in Fig. 1C. This was the only model used in our subsequent analyses.

The simplest possible RLDDM model is labelled RLDDM which we used as our base model for comparison. This is because it is essential to use the same type of data for all models, and so comparison with simpler models that only use choices but not reaction times is not quantitatively feasible.

### Parameter recovery

**Figure 2:**
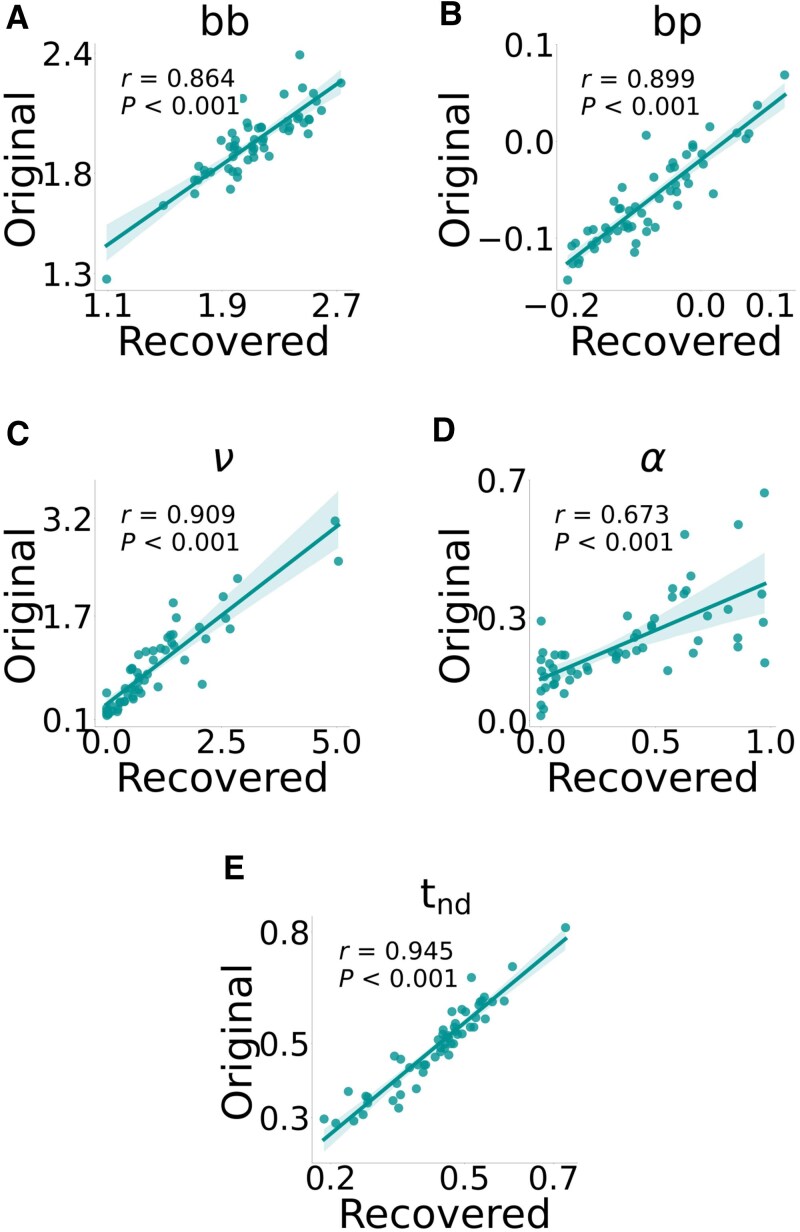
**Parameter recovery for the winning model.** Parameter recovery for each parameter (**A**) boundary separation base (bb), (**B**) boundary separation power (bp), (**C**) drift rate scalar (ν), (**D**) learning rate (α) and (**E**) non-decision time (t_nd_). Original parameters that were used to generate the synthetic data were plotted against the mean of the recovered parameter, with the shaded area being the 95% confidence interval of the regression line (solid line).

To ensure that the model parameters could be estimated from the behavioural data, we generated synthetic data using the winning model and the trial set-up (number of subjects, schedule, contingencies etc.). We then fitted our winning model to this generated data, successfully recovering all free parameters, Fig. 2.

### Decision-making analyses

The time evolution of the value estimates was expected to be biased by the quasi-randomized trial sequence and contingencies, which we aimed to remove by detrending the value estimates of the two depression groups. Hence, assuming that controls exhibited normal value time evolution behaviour, we took the mean value estimates at each trial from the control group and subtracted it from the two depression groups and took their mean values. It should be noted that the sequence of reward, neutral and loss trials was predetermined and the same for all subjects. In the description of the analyses that follow, we were only interested in the overall pattern of significant results, in relation to the study hypothesis.

Mean value signal estimates were calculated over the full 60 trials, and for exploratory analyses, for sections of the task (1–20, 21–40, 41–60). This was of interest because most learning occurs within the first 20 trials, and during the last 20 trials subjects are mostly executing their learnt behaviour. Hence, we divided the value estimates by trial type (reward and loss) as well as by choice options (high- and low-value options), giving us four different mean value estimates (four for each mean type) for each participant, and used one-sample *t*-tests to test hypotheses, also providing Cohen’s d effect sizes and Pearson correlations (effect sizes). Detrending the value estimates did not affect the correlation analyses, so are equivalent to calculating correlations using the original value estimates.

We categorized prediction errors into two types: positive, which indicates that the outcome was better than expected and negative, which implies the outcome was worse than expected. Since we are also interested in differentiating reward and loss trials, we subdivided these by trial type, calculating the mean across trials within each subgroup. Again, we took this mean across the full task and sections of the task, as with the value estimation analyses. This resulted in four types of prediction error (four for each mean type) for each participant, and we used two-sample *t*-tests to test hypotheses, also reporting Cohen’s d effect sizes and Pearson correlations (effect sizes).

We report both *P*-values from two-sample *t*-tests and Cohen’s *d* effect sizes for each model parameter. For the latter, we calculated the mean and standard deviation for each group over all samples, while for the former, we calculated the mean of each parameter for each participant over all samples.

### Neuroimaging analyses

Preprocessing of scans was done using Statistical Parametric Mapping 12 (SPM12).^55^ For each subject, we first realigned the images to the subject’s mean image then normalized these to the International Consortium for Brain Mapping space template for European brains. Finally, we smoothed these images with an 8 mm full width at half maximum Gaussian kernel.

An event-related design was used for the first-level analyses where event times were modelled with truncated delta functions and convolved with canonical haemodynamic response functions without time or dispersion derivatives. We individually tested our variables of interest (prediction error signals, decision time value signals, for both reward and loss trials) as parametric modulators with six covariates of no interest, rigid body motion realignment parameters and a constant term. Then, first-level ‘con’ images were used for second-level analyses to test for between-group differences (two-sample *t*-tests with Cohen’s d effect size) and for correlations with depression severity ratings, Hamilton Depression and Hamilton Anxiety. In all figures, significance was defined at a whole-brain level as a voxel threshold *P* < 0.05 with a simultaneous minimum cluster extent of >120 determined using a Monte-Carlo method.^56^ For illustration, the results were plotted using MRIcroGL^57^ using the relevant region of interest (ROI) mask, e.g. whole-brain analysis identified lateral orbitofrontal cortex differences when comparing bipolar depression with controls which was not present for unipolar depression and a region of interest was used to illustrate this.

Region of interest analyses^58^ were also conducted for *a priori* defined regions assuming significance as *P* < 0.05: the ventral tegmental area, nucleus accumbens, lateral orbitofrontal cortex, dorsal anterior cingulate, hippocampus, amygdala and hypothalamus. Masks for the ventral tegmental area and nucleus accumbens were defined as spheres with 8 mm radii and centres (0, −21, −13) and (6, 9, −4), respectively, estimated *a priori* from Mai’s high resolution anatomical atlas.^59^ The mask for the bilateral hippocampus was obtained from the Harvard-Oxford atlas from FMRIB Software Library^60^ at 50% threshold, the mask for the amygdala was taken from the automated anatomical labelling atlas^61^ and the mask for the bilateral hypothalamus was obtained from the BioImage Suite Web 1.2.0 atlas.^62^

### Discrimination of depression types

Null hypothesis testing does not take account of individual patient differences. As an exploratory analysis we therefore used a combination of machine learning that optimally combines the region of interest signals, and within-study replication (cross-validation) to avoid biased inferences,^15^ using a support vector machine with a linear kernel as implemented in NeuroMiner.^63^ For this, we used the mean signals from each ROI (left and right separately) and from both analysis types, giving us a total of 17 features. No feature reduction techniques were used. To ensure our accuracy was close to the true accuracy we used 10-fold nested cross-validation (10 inner and outer folds) with hyperparameter tuning automated to avoid potential overfitting and a fixed random seed of 654.

## Results

### Behavioural

#### PVS and NVS between-group analyses

Comparing controls to the combined unipolar depression and bipolar treatment-resistant depression groups, across all trial types, there were significant differences for both drift rate scalar (*ν*) [*t* = 1.99, *P* = 0.05, 95% CI (0.0, 1.96), *d* = 0.50] and boundary separation power (bp) [*t* = −2.74, *P* = 0.01, 95% CI (−0.47, −0.07), *d* = −0.70]. The former was lower for depression, implying the rate of evidence accumulation was slower, and the latter was larger for depression, indicating that patients had a higher reaction time asymptote. The latter implies that, on average, the reaction time of the control group approached a lower value with time than for treatment-resistant depression, consistent with psychomotor slowing, see Fig. 1B. For loss trials, for both unipolar depression and bipolar depression groups compared with controls, there was evidence of significantly blunted negative prediction error signals and increased loss value signals (see Supplementary material). No other significant group-level differences were found for the behavioural modelling. Significant differences were mostly driven by abnormalities in the bipolar depression group (see Supplementary material).

#### PVS and NVS correlation analyses

Considering the combined depression group, there was a significant correlation between Hamilton depression scores and non-decision time (*t_nd_*) [*r* = 0.39, *P* = 0.02, 95% CI (0.06, 0.64)]. This means that increased severity of depression was associated with a wider non-decision time window, indicating slowing of the rate of information processing. Conversely though, the drift rate scalar (*ν*) correlated with increased depression severity [*r* = 0.35, *P* = 0.04, 95% CI (0.01, 0.62)], indicating an increased rate of evidence accumulation with Hamilton depression score. This implies that the slowed rate of information processing (non-decision time) was partly offset by an increased evidence accumulation rate (drift rate scalar), with the latter effect outweighed by the former, resulting in psychomotor slowing in depression Fig. 1B. Additionally, during loss trials, we found that more severe depression was associated with blunted positive prediction error signals, and blunted reward value signals were associated with higher Hamilton depression and anxiety ratings (see Supplementary material).

### Neuroimaging analyses

#### Between-group analyses

##### PVS reward trials—prediction error signals

**Figure 3:**
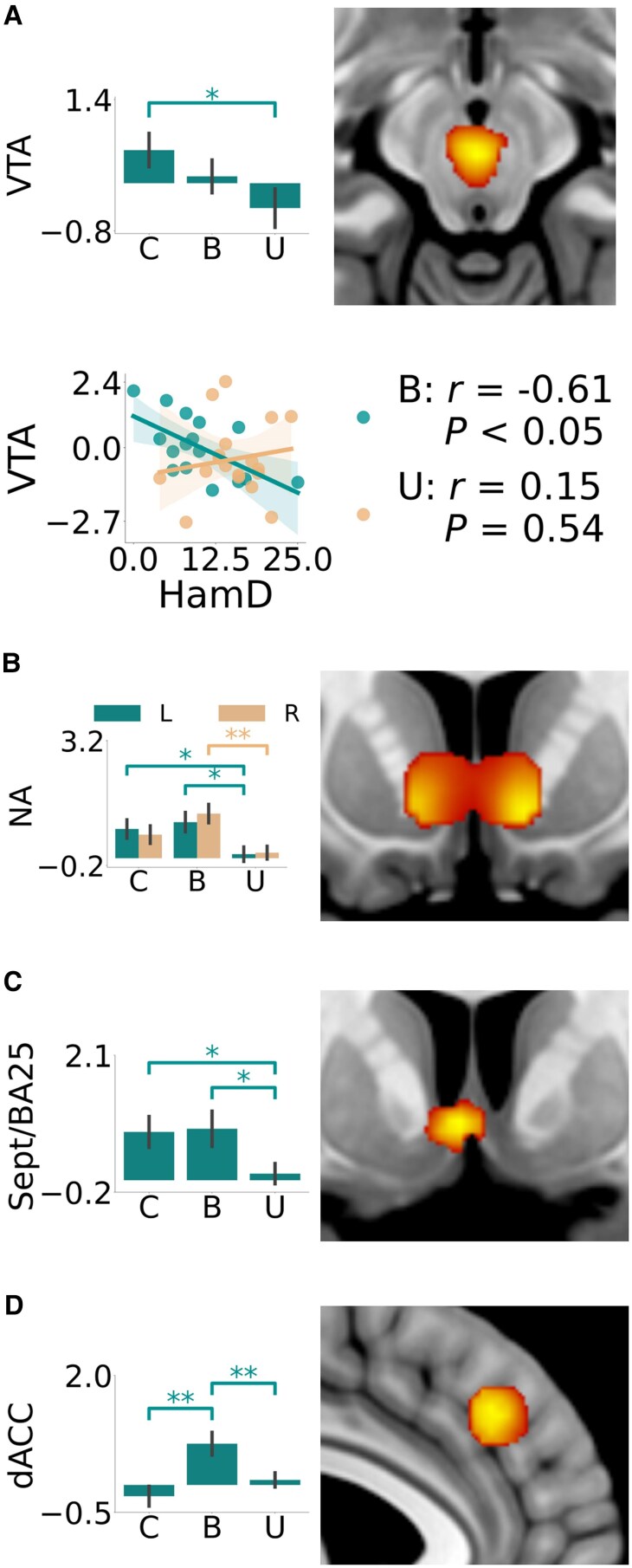
**Image analyses of the reward prediction error.** Reward prediction error signal at outcome time of the (**A**) ventral tegmental area (control > unipolar), (**B**) nucleus accumbens (activation of combined groups), (**C**) septum (control > unipolar) and (**D**) dorsal anterior cingulate, (control < bipolar). Region of interest analysis using the mean signal within each region is represented by bar plots along with a snapshot of the maps of the second-level voxel-by-voxel analysis captured at (**A**) *z* = −12, (**B**) *y* = 10, (**C**) *y* = 6 and (**D**) *x* = −4. Correlation between the mean ventral tegmental area signal and the Hamilton Depression scores is shown for the unipolar and bipolar groups separately. *P < 0.05, **P < 0.01. B = bipolar; C = control; dACC = dorsal anterior cingulate; HamD = Hamilton Depression Rating Scale score; U = unipolar; Hip = hippocampus; Hyp = hypothalamus; lOFC = lateral orbitofrontal cortex; L = left; NA = nucleus accumbens; R = right; Sept = septum; VTA = ventral tegmental area.

Comparing the unipolar depression group with controls, we observed blunting in the reward prediction signals in the ventral tegmental area [*t* = −2.29, *P* = 0.028, 95% CI (−1.83, −0.11), *d* = −0.75] (Fig. 3A), left nucleus accumbens [*t* = −2.10, *P* = 0.043, 95% CI (−1.35, −0.02), *d* = −0.69] (Fig. 3B) and septum [*t* = −2.21, *P* = 0.033, 95% CI (−1.34, −0.06), *d* = −0.72] (Fig. 3C). The bipolar depression group showed significantly increased reward prediction error signals in the dorsal anterior cingulate (Fig. 3D) compared with unipolar depression [*t* = 2.75, *P* = 0.010, 95% CI (0.17, 1.15), *d* = 0.94] and control groups [*t* = 3.51, *P* = 0.001, 95% CI (0.40, 1.51), *d* = 1.16].

##### PVS reward trials—detrended value signals at decision time

**Figure 4:**
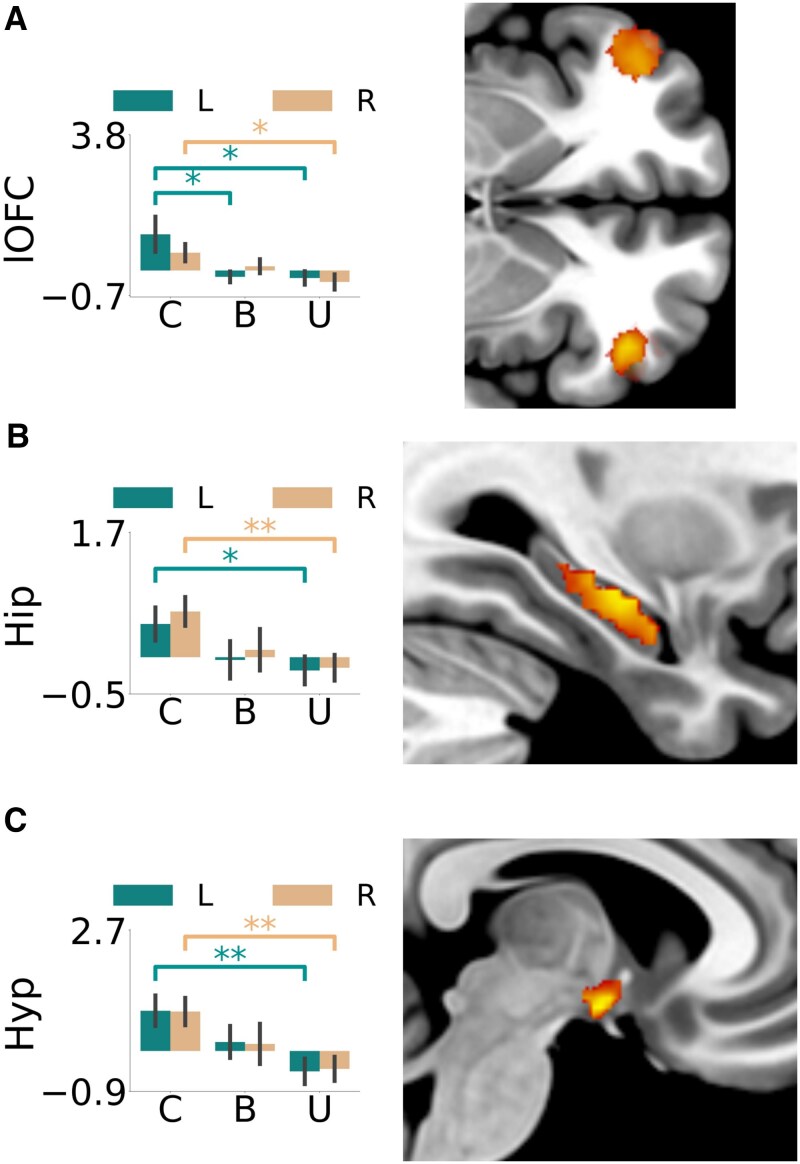
**Image analyses of the reward value.** Reward value (relative difference) estimates at decision time of the (**A**) lateral orbitofrontal cortex (control > unipolar), (**B**) hippocampus (control > unipolar) and (**C**) hypothalamus (control > unipolar). Region of interest analysis using the mean signal within each region is represented by bar plots along with a snapshot of the maps of the second-level voxel-by-voxel analysis captured at (**A**) *z* = −4, (**B**) *x* = 31 and (**C**) *x* = 6. Correlation between the mean ventral tegmental area signal and the Hamilton Depression scores is shown for the unipolar and bipolar groups separately. *P < 0.05, **P < 0.01. B = bipolar; C = control; U = unipolar; Hip = hippocampus; Hyp = hypothalamus; lOFC = lateral orbitofrontal cortex; L = left; R = right.

There was a significant decrease for reward value signals in the right lateral orbitofrontal cortex when comparing unipolar depression and control groups [*t* = −2.52, *P* = 0.016, 95% CI (−1.48, −0.16), *d* = −0.82] (Fig. 4A). For bipolar depression there was also a significant decrease compared with controls in the lateral orbitofrontal cortex [left, *t* = −2.15, *P* = 0.038, 95% CI (−2.31, −0.07), *d* = −0.71] [right, *t* = −2.22, *P* = 0.033, 95% CI (−2.34, −0.11), *d* = −0.73]. For unipolar depression compared with controls, there was a significant decrease for the hippocampus [left, *t* = −2.13, *P* = 0.040, 95% CI (−1.23, −0.03), *d* = −0.71] [right, *t* = −2.91, *P* = 0.006, 95% CI (−1.30, −0.23), *d* = −0.96] (Fig. 4B) and hypothalamus [left, *t* = −3.01, *P* = 0.005, 95% CI (−2.27, −0.44), *d* = −0.98] [right, *t* = −3.12, *P* = 0.004, 95% CI (−2.12, −0.45), *d* = −1.02] (Fig. 4C), which was not present for the bipolar depression group.

#### NVS loss trials—prediction error signals

**Figure 5:**
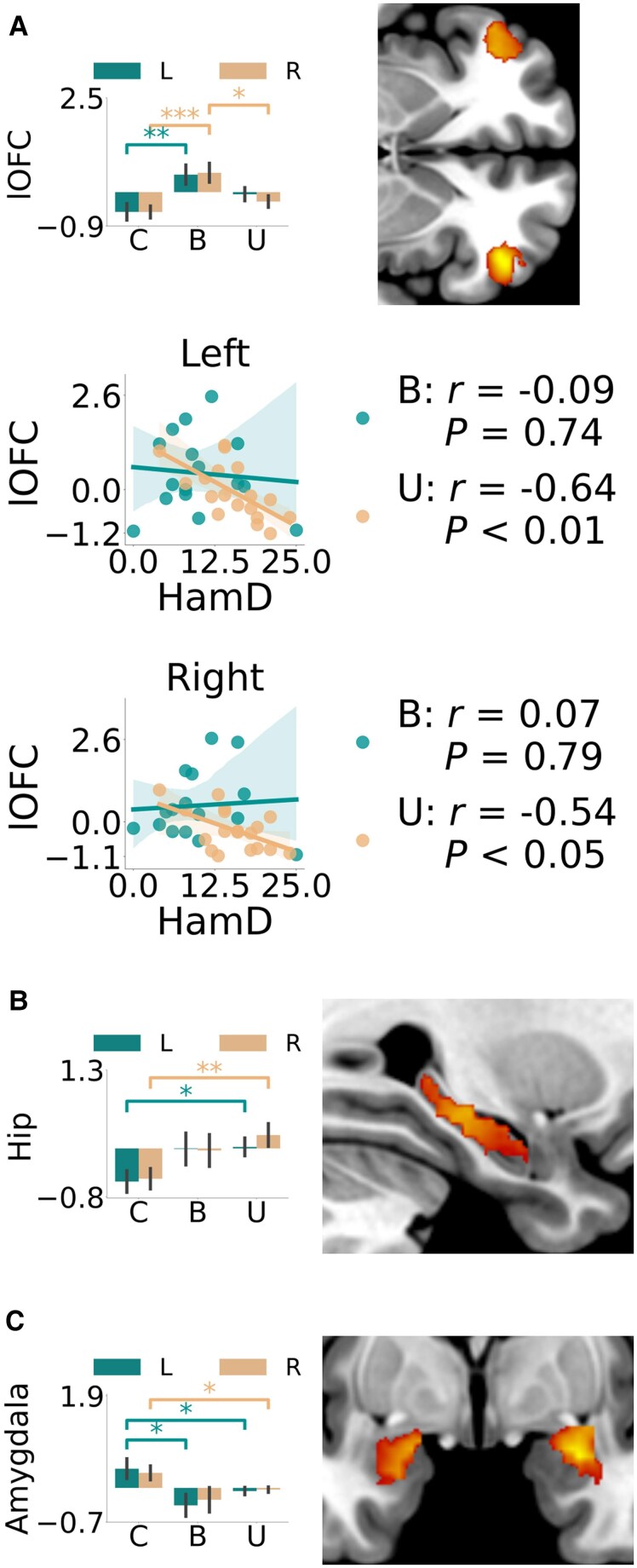
**Image analyses of the loss trials.** Loss prediction error signal at outcome time of the (**A**) lateral orbitofrontal cortex (control < bipolar) and (**B**) hippocampus (control < unipolar) as well as loss value (relative difference) estimate at decision time of the (**C**) amygdala (control > unipolar). Region of interest analysis using the mean signal within each region is represented by bar plots along with a snapshot of the maps of the second-level voxel-by-voxel analysis captured at (**A**) *z* = −4, (**B**) x = 28 and (**C**) y = −2. Correlation between the mean of the left and right lateral orbitofrontal cortex signals and the Hamilton Depression scores are shown for the unipolar and bipolar groups separately. *P < 0.05, **P < 0.01, ***P < 0.001. B = bipolar; C = control; U = unipolar; HamD = Hamilton Depression Rating Scale score; Hip = hippocampus; lOFC = lateral orbitofrontal cortex; L = left; R = right.

Comparing bipolar depression and control groups, there was a significant increase in the loss prediction error signals in the lateral orbitofrontal cortex [left, *t* = 2.96, *P* = 0.006, 95% CI (0.31, 1.66), *d* = 0.98] [right, *t* = 3.63, *P* = 0.001, 95% CI (0.46, 1.62), *d* = 1.21] (Fig. 5A). There was also an increase in loss prediction error signals found when comparing bipolar depression and unipolar depression groups in the right lateral orbitofrontal cortex [*t* = 2.64, *P* = 0.013, 95% CI (0.17, 1.35), *d* = 0.90]. Comparing the unipolar depression and control groups there was a significant increase in the hippocampus [left, *t* = 2.45, *P* = 0.019, 95% CI (0.10, 1.05), *d* = 0.80] [right, *t* = 2.87, *P* = 0.007, 95% CI (0.21, 1.23), *d* = 0.94] (Fig. 5B), which was not significantly different when comparing bipolar depression and control groups.

#### NVS loss trials—detrended value signals at decision time

Comparing unipolar depression and control groups, the amygdala showed a significantly decreased loss signal [left, *t* = −2.03, *P* = 0.05, 95% CI (−0.92, −0.00), *d* = −0.66] [right, *t* = −2.16, *P* = 0.038, 95% CI (−0.67, −0.02), *d* = −0.70]; see Fig. 5C. The bipolar depression group compared with controls showed a similar decreased signal in the left amygdala [*t* = −2.49, *P* = 0.018, 95% CI (−1.38, −0.14), *d* = −0.83].

#### PVS and NVS correlation analyses

For bipolar depression there was a significant negative correlation between the mean ventral tegmental area reward prediction error signal and Hamilton depression ratings (*r* = −0.61, *P* < 0.05), which was not present for unipolar depression (Fig. 3A), meaning more severe bipolar depression was associated with reduced ventral tegmental area reward prediction error signals. For unipolar depression there was a significant negative correlation between left and right average lateral orbitofrontal cortex loss prediction error signals and Hamilton depression ratings (left: *r* = −0.64, *P* < 0.01, right: *r* = 0.54, *P* < 0.05), which was not present for bipolar depression (Fig. 5A), meaning more severe unipolar depression was associated with lower loss prediction error signals within the lateral orbitofrontal cortex.

### Discrimination of depression types

**Figure 6:**
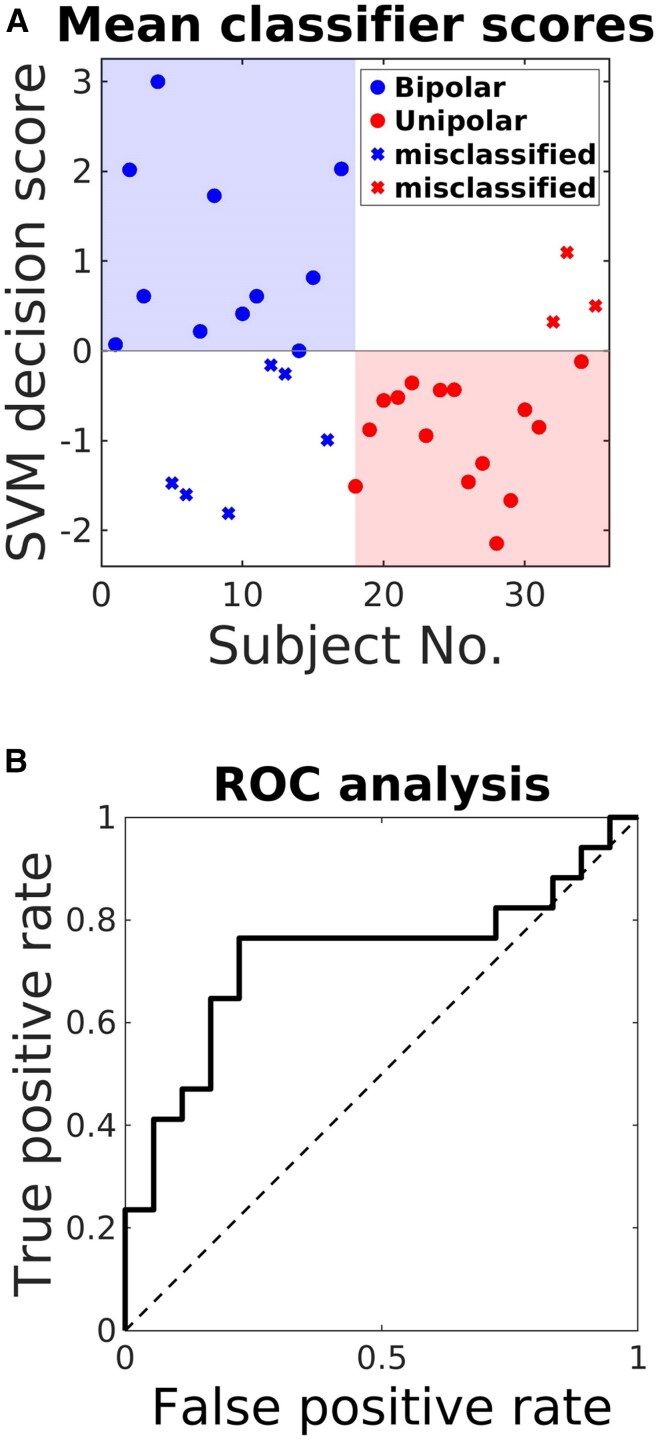
**Consistency analysis of activation patterns.** (**A**) Mean classification scores for each participant, with circles symbolize correctly classified and stars representing misclassified individuals. (**B**) The receiver operating characteristics (ROC) curve with area under the curve (AUC) of 0.73, 95% confidence interval (0.56, 0.90). SVM = Support Vector Machine.

The neuroimaging results are summarized in Table 2 which highlight a similar pattern of neurophysiological abnormality for treatment-resistant unipolar and bipolar depression compared with controls, with the exception of the reward prediction error signal in bipolar depression being abnormally increased. Comparing bipolar depression with unipolar depression, using all regions of interest from both the reward and loss neuroimaging analyses, we found the correct prediction of depression type for 74.3% of subjects (64.7% sensitivity and 83.3% specificity), with an area under the curve (AUC) of 0.73, 95% CI (0.56, 0.90); see Fig. 6. This means that even though treatment-resistant bipolar depression can be clinically indistinguishable from treatment-resistant unipolar depression, there is preliminary evidence depression types can be discriminated at an objective neural level.

**Table 2:** Summary of behavioural and neuroimaging findings

Behavioural analyses				
	Between-group analyses (versus controls)		Correlation analyses	
Unipolar and bipolar depression	Slowing indicated by decreased drift rate and increased boundary separation power		Positive correlation with non-decision time and drift rate with increased Hamilton score	
**Neuroimaging analyses**				
	**Reward Prediction Error**	**Reward value**	**Loss Prediction Error**	**Loss value**
Unipolar Depression	Decreased (nucleus accumbens, Brodmann area 25, septum, ventral tegmental area)	Decreased (lateral orbitofrontal cortex, hippocampus, hypothalamus)	Increased (hippocampus)	Decreased (amygdala)
Bipolar depression	Increased (dorsal anterior prefrontal cortex)	Decreased (lateral orbitofrontal cortex)	Increased (lateral orbitofrontal cortex)	Decreased (amygdala)

## Discussion

Using RLDDM modelling of behaviour with event-related model-based fMRI, we tested the null hypothesis that both unipolar and bipolar depressive illnesses show blunted PVS reward learning signals, and increased NVS loss avoidance learning signals, associated with psychomotor slowing. Consistent with this, we found abnormally slowed decision-making for both depression types, with individual patient RLDDM parameter estimates correlating with depression severity. For unipolar depression, brain activation patterns showed blunted outcome and value signals for positive feedback, and increased signals for negative feedback. In contrast to our findings for unipolar depression, bipolar depression was associated with preserved striatal reward prediction error signalling, and an absence of hippocampal and lateral orbitofrontal enhanced encoding of loss events, which was present in unipolar depression.

Psychomotor retardation in depression is an objective clinical sign comprising slowness in the initiation, execution and completion of actions, and slowness in speech, which patients may report as symptoms of slowed thinking and difficulty initiating and completing tasks.^64^ Psychomotor disturbance, particularly slowing, has long been recognized in mood disorders, as early as Kraepelin.^65^ More recently, psychomotor abnormalities have been discussed by Taylor and Fink,^66^ with rating scales proposed by Parker^36^ and Widlocher.^35^ These and other authors proposed that movement abnormalities are a core feature of moderate to severe mood disorders. In the present study, we used RLDDM to model psychomotor slowing then used model parameters as parametric modulators for the fMRI analyses.

Quantification by reaction time measurement allows objective identification of more subtle motor abnormalities in less severe illnesses. Studies using reward tasks and DDM to study unipolar depression have reported reproducible abnormalities in the drift rate^42,67-71^ and boundary separation^69,71^ parameters, the former being interpreted as indicating slower evidence accumulation and/or reduced memory capacity, the latter a need for more evidence accumulation before making a decision. Our reward task results are consistent with these studies. There are few unipolar depression studies on loss avoidance and these report inconsistent results.^42,72,73^ To our knowledge, no studies using reward or loss tasks have investigated current bipolar depression using DDM. Our study therefore replicates and extends previous work.

Regarding the positive valance system, a core feature of unipolar depression and bipolar depression is a subjective reduction in enjoyment of usually rewarding events, and a reduction in usual reward-seeking behaviour.^29^ The PVS^9,11^ processes reward information during reward learning and reward valuation. Consistent with an influential behavioural meta-analysis of unipolar depression and bipolar depression,^26^ we found that unipolar depression affects reward sensitivity rather than learning rate, finding abnormalities in drift rate and boundary separation power, and no significant difference in learning rate.

Neurosynth, a large-scale automated analysis of neuroimaging data identified 922 studies on reward events, reporting highly consistent activations in the bilateral nucleus accumbens, rostral-subgenual anterior cingulate and ventral tegmental area,^74^ consistent with the results of our study for control subjects. Although the ventral tegmental area is considered small, many previous studies have demonstrated that detection of brain activity is possible.^75–81^ Neuroimaging studies of unipolar depression have consistently reported blunted reward signals: blunted reward outcome signals,^13,16,19,20,23,25^ blunted reward prediction error signals,^14,15,17,18,21,22^ blunted reward anticipation signals^16,20,24^ and blunted reward value signals,^14^ consistent with a behavioural study^13^ and a behavioural meta-analysis on reinforcement learning in depression.^26^ Our results for unipolar depression and reward learning are consistent with this literature and the conclusions of a recent review.^27^

In contrast to unipolar depression, the recent review concluded that studies on bipolar depression have not reported consistently blunted striatal responses to rewards,^27^ and a recent experimental study reported no reduction in striatal reward prediction error signals in bipolar depression.^28^ Consistent with this, we did not find a blunted striatal prediction error signal in GAP patients with bipolar depression in long-term follow-up.

A number of studies of bipolar depression^27^ have reported increased left lateral orbitofrontal cortex activation during reward anticipation, testing different non-model-based hypotheses compared with our present study. Instead, we tested for a significant correlation between the reward value of the chosen stimulus inferred by modelling of decisions and response times, and anticipatory neural activity. We found blunted encoding of reward value signals in the lateral orbitofrontal cortex, bilaterally.

Regarding the negative valance system, increased attention to aversive events such as loss is emphasized in cognitive behavioural concepts of depression. The NVS^9,10^ processes information on aversive events including loss. Following Pessiglione *et al*.,^47^ we used a behavioural loss avoidance task for learning to avoid loss events, allowing modelling of loss prediction error and loss value signals during loss avoidance, independent of reward prediction error and reward value signals. As cognitive behavioural theories and treatments of depressive illness focus on abnormal responses to loss events in depression,^29^ it’s perhaps surprising that few event-related neuroimaging studies of unipolar depression and bipolar depression have investigated loss events including loss avoidance learning during current illness.

Deakin and Graeff^82^ reported an extensive and influential review of preclinical studies on animal models of human depressive illness using aversive learning, making predictions for humans with unipolar depressive illness, and highlighting as a prediction abnormal hippocampal function in humans. In the present study, we tested whether unipolar depression and bipolar depression were associated with abnormal loss prediction error signals and abnormal loss value signals. We found that the loss prediction error signal was increased in the hippocampus in unipolar depression compared with controls, but not in bipolar depression. In contrast, the loss prediction error signal was increased in the lateral orbitofrontal cortex in bipolar depression compared with controls, but not in unipolar depression. However, for both unipolar depression and bipolar depression, there was significantly decreased loss value signal encoding in the amygdala. This indicates that our results for unipolar depression are largely consistent with Deakin and Graeff’s predictions, but bipolar depression has a somewhat different pattern of abnormalities.

Kringelbach and Rolls^31^ reviewed orbitofrontal cortex function, concluding that the medial orbitofrontal cortex monitors rewarding events, whereas the lateral orbitofrontal cortex monitors non-reward (failure to achieve a reward when expected) and aversive (e.g. loss) events. Rolls^32,33^ highlighted the potential function of the lateral orbitofrontal cortex in depression, also reflecting its involvement in prediction error signalling. Consequently, the lateral orbitofrontal cortex may be essential for learning from negative outcomes, influencing decision-making. In the context of depression, this link might be disrupted^33^ and lead to non-optimal learning and decision-making. Our results for the PVS and NVS are consistent with this, also highlighting differences between unipolar depression and bipolar depression.

Clinically, unipolar depression can be indistinguishable from bipolar depression if a clear history of past mania or hypomania is unavailable, so there is a need for objective ways to discriminate these depression types.^27^ Whilst both illnesses had a similar overall pattern of neural abnormalities there were notable differences, particularly for the hippocampus, lateral orbitofrontal cortex and striatum. In an exploratory analysis we found it possible to predict unipolar depression from bipolar depression for individual patients with an accuracy of 74.3%, although independent studies are needed to confirm this finding.

Previous studies have reported prediction of unipolar versus bipolar depression using machine learning with different data types. These have included functional connectivity^83^ with 87.5% accuracy, tract-based spatial statistics^84^ with 73.65% accuracy, a large study of adolescents using questionnaire data^85^ reporting 80%–95% accuracy and blood biomarker data^86^ with an area under operating curve of 0.69. However, to our knowledge, no previous study has reported using fMRI for predicting depression types.

A strength of our model-based fMRI study is that we recruited currently ill patients with moderate severity treatment-resistant illnesses in long-term follow-up by GAP services. We have reported both a very large study on healthy volunteers and mild non-treatment-resistant depression,^22^ and independent studies on patients with moderate severity treatment-resistant illness in long-term GAP follow-up.^14,15,18^ In our experience, the latter have far slower recruitment, yet the relevance of the former to allow inferences about typical GAP patients remains unclear. For example, model parameter estimates and neural encoding of signals often correlate with illness severity, which may explain null results in large studies of mild illness, yet significant results can be found in smaller studies of patients with significant illness. Here, we were able to reject null hypotheses and predict depression type. Limited numbers of subjects primarily affects instances in which the null hypothesis was not rejected, e.g. it is possible that a larger bipolar depression group might allow detection of a slightly blunted striatal reward activation signal, although independent studies have also reported a lack of consistent striatal reward signal blunting in bipolar depression,^19^ in contrast to most studies of unipolar depression. Nevertheless, the relatively small group sizes highlights a need for independent replication of these results using a larger group of patients. A potential limitation was that the volume of the hippocampus has been reported to be smaller in both unipolar^87^ and bipolar^88^ depressions. To test whether this could have affected our results, we reduced the volume of our hippocampus ROI about 5%,^87^ but observed no significant difference to our findings. Another a potential limitation might have been lack of performance-related payments, but many previous studies^14,15,17,21,22^ have demonstrated the feasibility of using binary outcome feedback.

## Conclusion

Clinically, bipolar depression may often be unresponsive to antidepressant medication in contrast to unipolar depression, suggesting a different pattern of neural abnormalities.^4^ We found that different depression types were associated with different patterns of neural abnormalities, allowing objective discrimination. Further independent studies are required, focusing on loss avoidance learning in unipolar depression, and reward learning and loss avoidance learning in bipolar depression, with particular reference to the hippocampus, lateral orbitofrontal cortex and striatum, in patients who are ill at the time of study and also treatment resistant. Severe and enduring psychiatric illnesses are associated with considerable disability,^1^ a substantial reduction in average life expectancy^2^ and are relatively common, so further studies are indicated.

## Supplementary Material

awaf280_Supplementary_Data

## Data Availability

Data are available from the corresponding author on reasonable request.
