# Supplementary material for: Treatment-resistant recurrent unipolar and bipolar depression: associative learning abnormalities
Source: Brain. 2025 Aug 4;148(10):3705–17. doi: 10.1093/brain/awaf280 (PMC12493050; doi:10.1093/brain/awaf280)
Supplement: awaf280_Supplementary_Data [file awaf280_supplementary_data.pdf]

Supplementary Material Results

PVS and NVS Between group analyses

Comparing controls to bipolar depression negative prediction error was significantly blunted considering all loss trials (t=-2.79, p=0.008, 95% CI [-0.18, -0.03], d=-0.93). However, when controls were compared to unipolar depression, significance of blunted negative prediction error was only observed for the first 20 loss trials (t=-2.48, p=0.02, 95% CI [-0.20, -0.02], d=-0.81).

Considering loss trials, comparing controls to bipolar, we found that the value estimate of the higher value option was significantly increased for trials 1-20 (t=3.08, p=0.007, 95% CI [0.02, 0.10], d=0.71) and that the value estimate of the lower value option was blunted for trials 21-40 (t=-2.53, p=0.02, 95% CI [-0.12, -0.01], d=-0.58). On the other hand, comparing controls to unipolar depression and considering all loss trials we observed a significantly blunted value estimate of the lower value option (t=-2.08, p=0.05, 95% CI [-0.13, 0.00], d=-0.47).

PVS and NVS Correlation analyses

Considering the combined depression group and uuring the loss trials 1-20 we found significant correlation between depression severity (Hamilton depression and anxiety ratings) and positive prediction error (r=-0.36, p=0.037, 95% CI [-0.62, -0.02]), indicating that depression severity is associated with blunting. Also, during loss trials 21-40 there was a statistically significant correlation between depression severity and negative prediction error (r=-0.47, p=0.005, 95% CI [-0.70, -0.16]), also implying blunting for higher depression severity.

The combined depression group during the first 20 reward trials, showed a significant correlation between Hamilton anxiety rating and the value estimate of the lower value option (r=-0.38, p=0.03, 95% CI [-0.64, -0.05]), indicating that higher anxiety is associated with lower values. Also, during loss trials 21-40 Hamilton depression severity scores showed significant correlation with the value estimate of the lower value option (r=0.34, p=0.05, 95% CI [0.01, 0.61]), meaning that higher depression severity is associated with increased values.
